# An Artificial Intelligence–Based App for Self-Management of Low Back and Neck Pain in Specialist Care: Process Evaluation From a Randomized Clinical Trial

**DOI:** 10.2196/55716

**Published:** 2024-07-09

**Authors:** Anna Marcuzzi, Nina Elisabeth Klevanger, Lene Aasdahl, Sigmund Gismervik, Kerstin Bach, Paul Jarle Mork, Anne Lovise Nordstoga

**Affiliations:** 1 Department of Public Health and Nursing Norwegian University of Science and Technology Trondheim Norway; 2 Department of Physical Medicine and Rehabilitation St. Olavs Hospital Trondheim University Hospital Trondheim Norway; 3 Unicare Helsefort Rehabilitation Center Rissa Norway; 4 Department of Computer Science Norwegian University of Science and Technology Trondheim Norway; 5 Department of Neuromedicine and Movement Science Norwegian University of Science and Technology Trondheim Norway

**Keywords:** low back pain, neck pain, self-management, smartphone app, process evaluation, focus group, focus groups, musculoskeletal, mHealth, mobile health, app, apps, applications, usage, interview, interviews, qualitative, engagement

## Abstract

**Background:**

Self-management is endorsed in clinical practice guidelines for the care of musculoskeletal pain. In a randomized clinical trial, we tested the effectiveness of an artificial intelligence–based self-management app (selfBACK) as an adjunct to usual care for patients with low back and neck pain referred to specialist care.

**Objective:**

This study is a process evaluation aiming to explore patients’ engagement and experiences with the selfBACK app and specialist health care practitioners’ views on adopting digital self-management tools in their clinical practice.

**Methods:**

App usage analytics in the first 12 weeks were used to explore patients’ engagement with the SELFBACK app. Among the 99 patients allocated to the SELFBACK interventions, a purposive sample of 11 patients (aged 27-75 years, 8 female) was selected for semistructured individual interviews based on app usage. Two focus group interviews were conducted with specialist health care practitioners (n=9). Interviews were analyzed using thematic analysis.

**Results:**

Nearly one-third of patients never accessed the app, and one-third were low users. Three themes were identified from interviews with patients and health care practitioners: (1) overall impression of the app, where patients discussed the interface and content of the app, reported on usability issues, and described their app usage; (2) perceived value of the app, where patients and health care practitioners described the primary value of the app and its potential to supplement usual care; and (3) suggestions for future use, where patients and health care practitioners addressed aspects they believed would determine acceptance.

**Conclusions:**

Although the app’s uptake was relatively low, both patients and health care practitioners had a positive opinion about adopting an app-based self-management intervention for low back and neck pain as an add-on to usual care. Both described that the app could reassure patients by providing trustworthy information, thus empowering them to take actions on their own. Factors influencing app acceptance and engagement, such as content relevance, tailoring, trust, and usability properties, were identified.

**Trial Registration:**

ClinicalTrials.gov NCT04463043; https://clinicaltrials.gov/study/NCT04463043

## Introduction

Low back pain and neck pain are the main causes of disability worldwide [1]. Up to 30% of patients with acute or recurrent disability develop persistent pain [2,3]. Patients with persistent pain are often work-disabled and might need specialist assessment, which further increases health care and societal costs [4]. Given the highly prevalent and costly nature of low back and neck pain, enabling patients to self-manage constitutes an important strategy for reducing the individual and societal burden.

Self-management is commonly defined as an individual’s ability to actively monitor own health condition, adapt to physical and psychological demands, and implement lifestyle changes [5]. While self-management is endorsed in clinical practice guidelines to manage musculoskeletal pain [6], self-management support offered in clinical practice, for example, primary and specialist care, remains suboptimal [7,8]. Digital interventions such as smartphone apps can be a viable mode for delivering self-management support as an add-on to usual care due to their accessibility and possibility of making evidence-based advice easily available to patients.

We recently reported results from a randomized clinical trial (RCT) testing the effectiveness of an artificial intelligence (AI)–based self-management app (selfBACK) as an adjunct to usual care for patients with low back and neck pain in specialist care [9]. The app uses the case-based reasoning methodology, which is a branch of knowledge-driven AI [10] providing individually tailored self-management recommendations to users. Although individual tailoring is considered as an important feature for engagement in self-management interventions [11], the RCT did not show the SELFBACK app to be more effective than usual care alone or a web-based self-management intervention in improving self-reported musculoskeletal health. The aim of this study was to explore patients’ engagement and experiences with the selfBACK app and specialist health care practitioners’ views on adopting such digital self-management tools in their clinical practice.

## Methods

### Study Design and Context

This study is a process evaluation carried out in parallel with the RCT [9]. The qualitative part of the study is reported according to the consolidated criteria for reporting qualitative research (COREQ) [12].

Recruitment for the RCT took place at the multidisciplinary outpatient clinic for back-, neck-, and shoulder pain at St Olavs Hospital, Trondheim, Norway. Patients with low back and neck pain who were referred and on a waiting list for a consultation at the clinic were invited to the study via SMS text message. Interested and eligible patients were subsequently randomized to (1) the selfBACK app adjunct to usual care (99/294, 33.7%); (2) the e-Help (a self-management website) adjunct to usual care (98/294, 33.3%); and (3) usual care only (97/294, 33.0%). Usual care consisted of a waiting period of approximately 6-8 weeks before a consultation, including a clinical examination, followed by recommendations for suitable treatment. The recommendations could vary from no further treatment and adjusted recommendations for primary care treatment to outpatient multimodal rehabilitation or referral for surgery.

The process evaluation consisted of descriptive data analytics on app usage and semistructured interviews involving patients allocated to the selfBACK intervention. In addition, health care practitioners at the outpatient clinic were invited to participate in focus group interviews with the purpose of exploring their views on adopting digital tools for self-management support in their clinical practice. While health care practitioners were aware of the trial, they were not provided any specific instructions in relation to the trial conduct.

### SELFBACK App as an Adjunct to Usual Care

**Figure 1:**
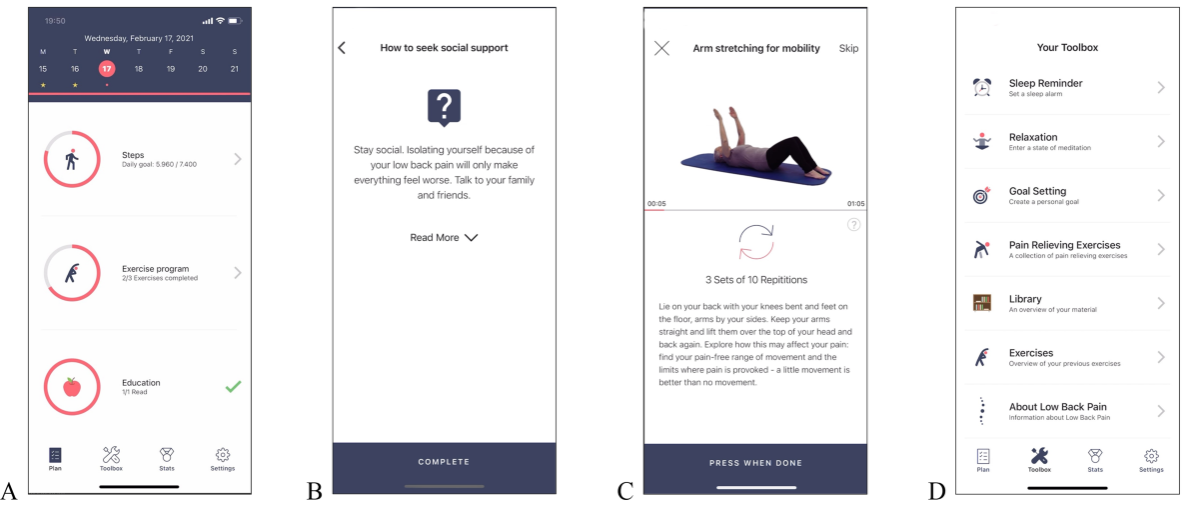
SELFBACK app screen views. (A) Home screen containing the 3 main components of the app, that is, steps achieved, exercise completion, and educational messages read. (B) Example of an educational message. (C) Example of exercise. (D) Toolbox screen containing additional resources.

The SELFBACK intervention was developed using intervention mapping [13] and underwent iterative pilot-testing before the final version was released [14,15]. The SELFBACK is an AI-based self-management app that provides users with weekly and individually tailored plans encompassing physical activity recommendations, strength and flexibility exercises, and educational messages (updated daily). In addition, the app contains a toolbox, which is a static component of the app containing, for example, goal-setting tool, mindfulness audios, pain-relieving exercises, and sleep reminders that patients can access at their own convenience [16] (Figure 1). The tailoring of patient recommendations delivered via the app relies on the application of case-based reasoning [10], a knowledge-driven AI methodology. In this methodology, knowledge from previous similar successful patient cases is reused to offer patient-centered and tailored recommendations. Thereby, new and similar patient cases receive recommendations based on what has or has not been successful in previous patient cases [17]. The AI system uses weekly reports (eg, symptom progression) and information collected through the app (eg, exercise completion and number of steps) to personalize the self-management recommendations. Patients can collect badges and rewards within the app by adhering to weekly recommendations. Push notifications are triggered by patients’ self-management behavior (eg, completion of exercises) and sent via the app to motivate and reinforce the desired self-management behavior.

The selfBACK app was offered as an adjunct to usual care. Patients randomized to the SELFBACK group were sent an SMS with a link to download the app. They were also provided with an installation guide and contact information of the research team if they had any access issues. Instructions on how to use the app and its content was provided within the app and patients had unrestricted access throughout the 6-month study period.

### App Usage Data Analytics

Data on app usage consisted of information about number of weekly plans generated, number of app access per week, and specific content visited. For weekly plans to be generated, the patient needed to access the app and complete the weekly short-tailoring questionnaire (eg, questions on pain intensity, self-efficacy level, and fear avoidance level). The number of weekly plans generated was used to dichotomize patients into moderate or high users and low users as basis for a purposive recruitment for interviews. Moderate or high use was defined as generating at least 6 plans during the first 12 weeks after first access of the app, while low use was defined as generating less than 6 plans as described previously [9]. This information together with the number of app access per week was retrieved from the back end of the AI system, which has information when users actively interact with the app (completing exercises, tailoring sessions, or similar). Information about number of days a specific content was visited per week (eg, exercises, educational component, and toolbox) was retrieved from Matomo [18], a free and open-source software that records whenever a user accesses a screen of the app.

### Interviews With Patients and Health Care Practitioners

A purposive sample of 15 patients was contacted by phone and invited for interviews according to their app usage (ie, number of weekly plans generated). Of these, 3 declined participation and 1 did not answer. A total of 11 patients were interviewed (aged 27-75 years, 8 female), of whom 7 were moderate or high app users and 4 were low users.

Health care practitioners from the multidisciplinary outpatient clinic (n=11) were informed about the study during a regular staff meeting and invited to participate in focus group interviews. Overall, 9 health care practitioners expressed an interest in participating and were included. Two focus groups were formed based on the role the health care practitioners had at the clinic. One focus group (focus group 1) included 3 physiotherapists and 2 social workers (aged 32-51 years, 4 female) with 5-13 years of working experience at the outpatient clinic. The other group (focus group 2) included 4 physicians (aged 32-42 years, 3 female) with working experience at the outpatient clinic ranging from 1 week to 7 years.

The interviews with patients and health care practitioners were performed by a research assistant with prior experience of conducting qualitative interviews and no prior relationship with patients or health care practitioners. Patients were interviewed individually via Skype between January and February 2021. The interview guide was semistructured and developed using the Normalization Process Theory [19,20]. The questions included background information, the motivation to join the study, how pain was managed before the study, what facilitated or hindered the use of the app, how the app was integrated in daily life, future intentions to use the app, and general thoughts about self-management. Questions were adapted when needed and follow-up questions added where appropriate. Each interview lasted approximately 45 minutes and was recorded with the patient’s permission. One interview was repeated due to failure of the recording.

The focus groups took place digitally via the Zoom (Zoom Video Communications) platform in February 2021. Prior to the focus groups, health care practitioners were provided with an overview of the selfBACK app by the research team and access to the app. They were asked about their initial impressions of the selfBACK app, their views on digital tools to support self-management, whether and how they would use them in clinical practice, what potential benefits and risks such tools entailed, whether they believed that using them could affect their professional autonomy, and whether such digital tools could be trusted. Each focus group lasted approximately 90 minutes and was facilitated by 2 research assistants (one acting as an observer). At the end of each focus group, the 2 researchers exchanged experiences on the interaction among health care practitioners and these were annotated. Both interviews and focus groups were audio recorded with the permission of all participants and transcribed verbatim. Data were de-identified during transcription and used thereafter for data analysis.

### Data Analysis

Baseline characteristics of all patients were reported descriptively. Interviews and focus groups data were analyzed using thematic analysis [20]. First, ALN and AM read and coded the interview transcripts with the support from the research assistant who transcribed the interviews to ensure that coding was reflective of the material. The codes were then grouped into themes by a process of constantly deliberating their content and boundaries, resulting in 2 coding trees for the patients and health care practitioners, respectively. These were subsequently discussed with 2 researchers in the team (NK and LA) who had read all the interview transcripts. As the 2 coding trees were found to largely contain complementary themes, they were combined before writing up the results. Quotations were added to either exemplify or nuance the analytic text.

### Ethical Considerations

The RCT was registered in ClinicalTrials.gov (NCT04463043), and the protocol, including the description of the process evaluation, was published [21]. Ethics approval was granted by the Regional Committee for Medical and Health Research Ethics in Central Norway (reference 64084) and the Norwegian Medicines Agency (reference 20/10329-10). All patients provided written informed consent before entering the study.

Health care practitioners were provided with oral information about the study and verbal informed consent was obtained from them before the focus groups were conducted.

## Results

### App Usage

The demographic and clinical characteristics of the 99 patients allocated to the selfBACK intervention stratified by app usage are shown in Table 1. Overall, patients’ characteristics were similar across groups, although a greater proportion of patients who never accessed the app or who were low users reported having daily pain as well as having both neck and back pain compared with moderate or high users who reported pain less frequently and predominantly pain at the lower back (Table 1).

**Table 1:** Demographic and clinical characteristics of the 99 patients allocated to SELFBACK intervention, stratified by app usage.

		Never accessed app	Low usage group^a^	Moderate or high usage group^b^
Participants, n (%)		29 (29.3)	32 (32.3)	38 (38.4)
Age (years), mean (SD)		52.3 (11.5)	47.8 (14.2)	50.8 (16.4)
Women, n (%)		17 (58.6)	19 (59.4)	24 (63.2)
**Education (years), n (%)**				
	>12	16 (26.2)	19 (31.2)	26 (42.6)
	10-12	9 (32.1)	10 (35.7)	9 (32.1)
	<10	4 (40.0)	3 (30.0)	3 (30.0)
Full-time or part-time employment, n (%)		19 (65.5)	25 (78.1)	26 (68.4)
Married or living with partner, n (%)		20 (69.0)	23 (71.9)	29 (76.3)
**Pain localization, n (%)**				
	Low back pain	13 (25.0)	16 (30.8)	23 (44.2)
	Neck pain	6 (24.0)	11 (44.0)	8 (32.0)
	Neck and low back pain	10 (45.5)	5 (22.7)	7 (31.8)
**Days with pain past year, n (%)**				
	≤30 days	3 (42.9)	1 (14.2)	3 (42.9)
	>30 days but not every day	7 (18.4)	10 (26.3)	21 (55.3)
	Every day	19 (35.2)	21 (38.9)	14 (25.9)
**Use of pain medication (days per week), n (%)**				
	None	8 (28.6)	9 (32.1)	11 (39.3)
	1-2 days	8 (40.0)	5 (25.0)	7 (35.0)
	3-5 days	7 (31.8)	6 (27.3)	9 (40.9)
	Daily	6 (20.7)	12 (41.4)	11 (37.9)
Musculoskeletal Health Questionnaire (score range 0-56), mean (SD)		30.9 (9.0)	28.4 (8.6)	30.8 (9.6)
Average pain intensity level past week^c^ (score range 0-10), mean (SD)		5.7 (2.4)	5.5 (1.6)	4.8 (2.0)
Worst pain intensity level past week^c^ (score range 0-10), mean (SD)		6.9 (2.3)	7.3 (1.6)	6.3 (2.1)
Health-related quality of life^d^ (score range 0-100), mean (SD)		52.7 (20.6)	56.3 (14.1)	57.5 (19.2)
Pain Self-Efficacy Questionnaire (score range 0-60), mean (SD)		37.8 (13.7)	36.5 (14.0)	39.3 (12.1)

^a^Low usage group comprises patients who generated less than 6 of 12 weekly plans.

^b^Moderate or high usage group comprises patients who generated 6 of 12 weekly plans.

^c^Measured by the Numeric Rating Scale.

^d^Measured by the Visual Analogue Scale in the EQ-5D.

**Figure 2:**
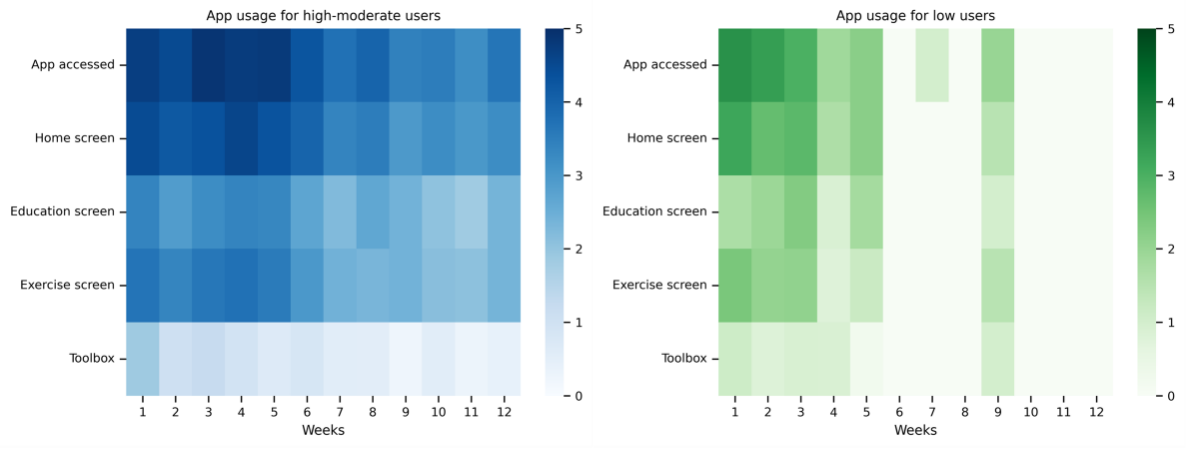
Average number of days each app component was visited in the first 12 weeks. Moderate or high users are shown on the left and low users are shown on the right. The bar to the right of each graph indicates the shading according to the average number of days each component was visited. App-accessed row shows any interaction with the app. Home screen, education screen, exercise screen, and toolbox rows represent the main components of the app shown in Figures 1A-D. Only those users who accessed the app at least once (n=70) are shown.

Figure 2 shows usage of each app components in the first 12 weeks stratified by usage group. Patients in the moderate or high usage group accessed the app, on average, most days of the week (ie, 4-5 days) for the first 5 weeks and somewhat reduced the frequency of weekly app access thereafter. The most visited content was the exercises, followed by the educational messages which were accessed, on average, more than once a week throughout the 12 weeks. The toolbox (ie, the static component of the app) was visited the least (Figure 2). Patients in the low usage group accessed the app, on average, fewer days per week from the beginning and mostly discontinued use after 5 weeks (Figure 2).

### Interviews With Patients and Health Care Practitioners

We found that the interviews with patients and health care practitioners generated valuable information on their overall impression of the app, its perceived value, and suggestions for future use.

### Theme 1: Overall Impression of the App

Patients discussed the interface and content of the app, reported on usability issues, and described their app usage.

### App Interface and Content

Most patients described the app’s user interface as simple and intuitive, with a user-friendly layout that was easy to navigate. Both patients and health care practitioners appreciated how the information was presented, for example, short and structured, and receiving a weekly plan was a convenient feature some patients valued.

You get a full program. You don’t have to make it yourself. You are reminded and guided through the exercises so that you do not have to think and remember and count.

Female, 50 years, low user

When describing the content of the app, patients in both usage groups talked extensively about the exercise component. Indeed, being instructed on specific exercises was one of the main motivations for joining the study for most, and the integrated exercise videos were a feature they highly valued. Many also described that while they were often familiar with the educational content in the app, this nevertheless served as valuable reminders in their daily life. However, being familiar with the exercises and the educational content in the selfBACK app also resulted in some patients becoming unmotivated. In addition, some patients found the app’s content to be tailored to their individual needs, while others believed that the content was of low relevance to them.

I thought that the exercises were very neutral, and I didn’t get an exercise that suited my injury.

Female, 46 years, low user

### Usability

Many patients in both user groups reported various technical problems when using the app. In the low usage group, some found the onboarding procedure burdensome and lost motivation to use the app. Several patients also had technical difficulties with step synchronization (ie, between the selfBACK app and health apps registering steps), which made them frustrated as notifications encouraging physical activity felt inappropriate when they had been active, and since they did not get validation when achieving their goals. In addition, some patients mentioned difficulties in scoring the weekly questions about their symptoms and function or not finding them relevant, although this issue did not prevent them from using the app. One patient also reported that the audio in the mindfulness section was difficult to hear due to a hearing impairment, and even though this feature felt relevant, they could not use it.

### Usage Behavior

When describing how they used the app, most patients in the high usage group said that they used it regularly at the beginning and then gradually discontinued use. Some reported that, over time, they felt less need to access the app content and register their activity, mainly since their pain symptoms subsided.

My back improved, and I don’t have the same need to use it and getting that recognition for progression and so on.

Male, 27 years, high user

Some patients commented that they performed the exercises in the app in combination with other exercises they already knew from before or with pain-relieving exercises in the toolbox (a static library of exercises within the app).

### Theme 2: Perceived Value

Patients and health care practitioners described the primary value of the app as providing reassurance to patients by offering trustworthy information, thereby empowering them to self-manage. They also described the potential of the app to supplement usual care.

### Providing Reassurance

Several patients described feeling reassured by the information in the app and experiencing exercises as manageable and not harmful.

These three things [components on the main page of the app] together give you some input at least during a period where you’re unsure of what you can do, because it hurts really bad. And then the app comes with a little advice: okay, even if you’re in pain then it doesn’t get worse or, yes, it’s unlikely to get much worse. And I believe that has helped.

Female, 54 years, high user

Most patients reflected on how recommendations encouraging activity in the selfBACK app also aligned with advice from chiropractors and physiotherapists they had consulted previously. As such, the coherence between the information in the app and health care personnel reassured them that the information and exercises could be trusted. On the contrary, 1 patient experienced that her health care practitioner did not endorse the advice provided by the app and, as a result, she discontinued using it, feeling insecure about its appropriateness for her situation.

I showed my therapist [chiropractor] the exercise, and he said right away: “you shouldn’t do that exercise. Because then you make it worse for yourself”.

Female, 46 years, low user

Health care practitioners underlined the need to present patients with reassuring language. The app was described as an opportunity to prevent patients from getting information from unreliable sources on the internet and provide patients with up-to-date, reliable, and consistent information that reinforced their message.

Knowing that the information is given via the health service can be reassuring, and they [the patients] may be more confident that it is nuanced and correct. They can go back and see “yes, that’s consistent with what the doctor said”.

Female, focus group 2

In line with this, some patients also described how their confidence in digital tools would increase if a health care practitioner or other trustworthy sources had developed or endorsed them in contrast with commercial parties.

One patient also described valuing how an app was made specifically for her health condition and that digital tools in general made her feel taken care of and included.

You can say that even if it’s a robot, just the fact that you get a message, one feels taken care of in some way. And feeling like you’re not alone in what you’re struggling with, the app becomes a symbol that there are many others who are struggling with it [musculoskeletal pain].

Female, 50 years, low user

### Empowering Patients to Self-Manage

Some patients described that using the app supported them on the road to becoming more active and that their confidence and thoughts about self-management increased while using it.

I’m just going to have to try on my own now. What’s working and what’s not working. It’s not that I’m afraid something’s dangerous anymore.

Female, 44 years, high user

Some also pointed out that although the information about self-management was perhaps well known to them, it nevertheless encouraged the thought of being able to act on one’s own.

It has helped to think a little more positively and, yes, that you can do a lot yourself. This kind of things you know deep down, but it’s about getting a little help to put your thoughts on the right track.

Female, 54 years, high user

Some patients described features such as reminders and activity tracking as positive influences on motivation to be active, and these also served as a reminder of how much they achieved. Health care practitioners similarly believed that digital tools such as the selfBACK app could help encourage patients’ active participation in rehabilitation. The interactive features (eg, goal setting) and accessibility of the app were also mentioned as elements that can promote and reinforce self-management behaviors*.*

A nice thing is that you could make your own personal goal […], you have to take a position on some questions. For example, you are asked a lot about this goal, whether it is realistic, how long it should last. So, you have to make some active choices.

Male, focus group 1

One physiotherapist described this active approach as taking responsibility instead of clientification, a point also reflected in the statements of many patients.

There is something that we hope [to achieve] in collaboration with the patient, which is to make them accountable, so that the patient sits in the driver seat.

Female, focus group 1

### Supplementing Usual Care

Both patients and health care practitioners believed that the selfBACK app could be a valuable supplement to usual care. When reflecting on how digital tools can help in taking responsibility for one’s health, several described it as a necessity and solution to the increasing pressure on health care services.

Adopting an app such as selfBACK in clinical practice was described by both patients and health care practitioners as valuable to compensate for current organizational constraints (eg, long waiting time for the first consultation at the clinic, short consultations, limited service for people living in remote areas, and as a supplement to the physiotherapy service). One physician added how being active in advance could be helpful during consultations since the patients would then find it easier to explain their difficulties. Another physician described how the app positively impacted patients’ health while awaiting health care assessment.

There is a long waiting time to get an appointment with us. Some patients have already gotten much better when they meet at the first appointment with me because they have started with physical activity and exercises on their own through the app […]. I think it is a great way to get them started.

Female, focus group 2

Others underlined how the app could also help maintain continuity for patients between consultations or cut down on the number of consultations, and some described the app as a way to support patients benefitting from less-intensive treatment. Therefore, the app was seen as a potential aid in allocating health care resources more appropriately to those needing it the most.

We have talked a lot about who is the right patient for us. And then we concluded that the so-called simple patients, those who can get help, for example, from an app by taking some steps in their lives that enable them to function, might not be the right patients for us. […] So, if we have a tool that we feel is good and can help some of these patients, it is fantastic. Then we handle the more complex cases, where an app is not sufficient.

Female, focus group 1

However, patients and health care practitioners emphasized that digital tools such as the selfBACK app should be regarded only as a supplement to usual care and not as a replacement. One patient explained how she believed that severe conditions should be ruled out first, which 1 physician also underlined when describing how many patients are not reassured by solely receiving information, for instance, when experiencing radiating pain. Another physician also described how normalizing pain in some, yet very few cases, can be inappropriate, and that the app should be combined with a health care consultation in such cases.

Several patients also underlined how their trust in and enthusiasm for technological tools did not imply that it could substitute the human contact offered by consultations with health care practitioners due to the value such interpersonal relationships represent. Similarly, the health care practitioners commented that they did not feel that the selfBACK app would interfere with their professional autonomy, seeing their role as essential.

Even if the app, based on how the patients respond to questionnaires, is customised and makes individual adaptations, it will never be able to do what a physician might do, see the patient in a larger perspective.

Female, focus group 2

### Theme 3: Suggestions for Future Use

Although patients and health care practitioners felt positive about the possibility of adopting tools such as the selfBACK app and making progress on their own, they addressed several aspects they believed would determine acceptance. Many suggestions for change aligned with the difficulties described in the overall impression of the app regarding usability and content.

Some health care practitioners mentioned that the start-up process should be made more efficient if patients were to adopt the selfback app. Although patients and health care practitioners highlighted the possibility of replacing exercises as a valuable feature, some patients wished for an opportunity to provide feedback or point out the issues they experienced. Some patients also felt that they would have benefitted from more instructions within the app, for example, describing the frequency and purpose of the exercises, a point also reflected on by some physiotherapists.

Physiotherapists also said that the extensive focus on exercises was less beneficial than instructing patients to find an activity they liked to start being active. In addition, they pointed out that some exercise descriptions potentially undermined the main message of the app that activity is not harmful by communicating the opposite impression.

It [the app] uses words like “careful, controlled movement”. Then you are communicating that you can potentially destroy something.

Female, focus group 1

Some physiotherapists and social workers also suggested that information on how other aspects, such as anxiety and depression, contribute to the feeling of pain should be highlighted within the app. In addition, 1 physiotherapist found the goal setting in the app so important to patients’ rehabilitation processes that they suggested that it should be made mandatory to fill it in to proceed further.

Health care practitioners also reflected on aspects facilitating implementation in clinical practice. One stated how it would be beneficial to refer patients to something specific, such as the app, instead of a general call for “being active.” Physiotherapists and social workers commented that having access to patients’ interaction with the app would enable them to integrate it into their clinical routine. This point was also reflected by a statement from a patient.

It’s nice if you have such an app, which you can choose to get and use yourself. Then you might get a follow-up with a doctor by phone or something like that asking: “What is the status now?”. And the doctor might also be able to see the updates in the app and what you have posted. It is a tool for both the doctor and the patient if both have access to the results of such an app.

Female, 59 years, low user

## Discussion

### Principal Findings

This study explored the engagement and experiences with an app-based self-management support system (selfback) for patients with low back and neck pain referred to specialist care, and health care practitioners’ views on adopting such digital tools in their clinical practice. Overall, patients’ experiences and health care practitioners’ perception of the app largely overlapped. Both had a positive attitude toward adopting app-based self-management support in this setting and saw a large potential in the selfback app to supplement usual care. Both described how the app can reassure patients by providing trustworthy information, thereby empowering them to take action on their own. Usability properties, content relevance, and the role of health care professionals were identified as important elements influencing acceptance and further engagement with the app.

While patients and clinicians were positive about the adoption of app-based self-management support for low back and neck pain as a supplement to usual care, the uptake of the intervention across patients enrolled in the RCT was relatively low. This somewhat differed from the SELFBACK trial in primary care, where nearly two-thirds of patients sustained use throughout 12 weeks [22,23]. Such differences might be partly explained by the onboarding procedures used, as well as by the study setting, that is, patients waiting for further consultation after referral to specialist care might be less prone or motivated to explore self-management interventions. Furthermore, low or nonusers in our study reported greater pain frequency and more widespread pain than moderate or high users. More burdensome health conditions, for example, having comorbidities or high symptom severity, have been suggested to be a barrier for engaging with self-management interventions [24,25]. Thus, understanding how the heterogeneity in clinical features might affect the uptake and engagement of self-management interventions should be explored further, particularly since greater symptom burden does not seem to modify the effect of such interventions [26-28].

Successful implementation of digital interventions relies, at least in part, on patients’ acceptance of the intervention. Patients indicated that factors promoting the app’s adoption included that it was easy to use, convenient, and provided structured and tailored information (eg, weekly plans). Furthermore, some patients described that knowing that the app was coming from a trustworthy source (ie, health care system or university) facilitated acceptance. On the contrary, technical difficulties, perceiving the content as irrelevant or not new, and lack of endorsement from the health care practitioner hindered some users from adopting the app. These elements are in line with existing acceptance models positing that perceived ease of use, perceived usefulness, and trusting beliefs in health care providers (or vendors) are, among other factors [29], significant predictors of behavioral intention in digital interventions [30,31].

Ensuring adequate engagement is a prerequisite for the effectiveness of app-based interventions [32]. Some patients described how notifications, activity tracking, and rewards helped them stay engaged with the app’s content, emphasizing the importance of interactive, tailored support for sustaining self-management behaviors [11]. Such reminders seemed particularly relevant in the first phase of use, as some patients reported not having the need to log their activities or getting the recognition of achievements once the symptoms subsided. This use pattern has been described in previous digital interventions for low back pain [22,25]. The fact that some patients perceived the app as a supporter and tailored to one’s individual needs suggests a form of therapeutic alliance with the digital interaction [33], which is an important enabler of self-management [24] and has been linked with increased engagement [34]. Conversely, perceiving the app as too general and irrelevant to one’s health condition, as reported by other patients, might prevent the establishment of such a bond [33]. Technological and human-like design features, for example, AI chatbot, avatars, social forums, and peer support, can potentially foster digital therapeutic alliance further [35,36], which could be interesting to explore in future developments.

While the selfBACK app was designed to be self-explanatory, some patients indicated the need for more instructions. This was partly reflected by the fact that the exploration of the app was mostly limited to the main components of exercises and physical activity (ie, step count). Other components in the toolbox (static component within the app) containing additional self-management resources (eg, goal setting, pacing, relaxation techniques, and mindfulness) were less explored, as reflected by the usage data and the interview data. Although suggestions to access these resources were somewhat integrated into the educational content, they were not very prominent in the design of the weekly plan algorithm compared with the exercises. This might have limited the exposure and practice of relevant self-management skills linked to the promotion of self-efficacy, in turn influencing long-term behavioral change [24]. A few patients also mentioned the necessity of customizing some elements within the app (beyond changing exercises) and the ability to provide feedback. This need for greater self-tailoring of the content aligns with the concept of autonomy support, whereby taking individual preferences into account and enabling patients’ perceived active control foster autonomous motivation, which is important for the maintenance of behavior change [37].

Offering a self-management app for patients on a waiting list for specialist care can be an easy and inexpensive approach to initiate cognitive and behavioral processes by providing evidence-based and tailored content. Some patients described being reassured by the educational content and exercises and developing greater awareness and confidence about the possibility of self-managing while using the app. As such, the app can increase patients’ feeling of empowerment, which is important to achieve competence to manage pain and enable lifestyle changes [38]. The clinical value of embedding a self-help intervention in this phase was further highlighted by health care practitioners who stated that priming patients with such content would enhance patient-clinician communication, thus facilitating shared decision-making during the clinical encounter. However, both patients and health care practitioners often mentioned the need for clinical involvement to enable engagement with self-management advice, mostly due to diagnostic uncertainty in this patient group. Previous research has shown that health care professional support, even when remote or minimal, can increase the effectiveness of self-management interventions [39]. Thus, combining digital and human support could be a useful approach to enhance adoption of self-management, particularly in the specialist health care setting with long waiting time.

Both patients and health care practitioners widely emphasized the necessity for taking responsibility for one’s own health conditions, indicating that digital interventions such as the SELFBACK app hold a large potential in mitigating current health care shortage challenges. However, while digital interventions are useful and wanted by many patients, our findings suggest that not all patients can benefit from such interventions. Since the patient group in this study is highly heterogeneous and pain management styles and preferences vary, further research should look into how to further optimize tailoring of self-management support to increase patients’ feeling of relevance and usefulness over time. In addition, patients’ needs, abilities, and preferences for autonomy should be considered when implementing digital interventions within the health care setting [40]. This should come with the awareness that assuming patients’ responsibility for self-management could lead to stigmatization of some patients, potentially those with higher needs for health care services [41].

### Strengths and Limitations

While our findings might not be generalizable to other contexts (eg, different health care systems) or patient groups with other chronic health challenges, they nonetheless provide useful insights into patients’ and health care practitioners’ experiences with digital self-management interventions. Since back pain complaints are one of the main causes of years lived with disability worldwide [42] and practitioners’ acceptance of app-based interventions has been recognized as a global tendency [43], the need and value of digital self-management support transcend the regional setting of this study.

A strength of this study was that researchers from different backgrounds, that is, physiotherapy, medicine, anthropology, and exercise physiology read the interviews, and results were discussed thoroughly among them. Another strength was the inclusion of patients with different levels of app usage (ie, the number of plans generated), ensuring a balanced view of patients’ experiences with the app, including those who might have been less satisfied with it. In addition, data on how much the users accessed different content in the app were available for all patients allocated to the SELFBACK app. Integrating the views of health care practitioners with patients’ experiences allowed a better understanding of acceptability and needs from both sides, which are important for future implementation. However, some limitations need to be considered. Although health care practitioners were invited to get acquainted with the app for some weeks prior to the focus groups in addition to receiving an overview of its functionality, only a few tried the app and were familiar with the entire content. A greater firsthand experience could have increased the specificity of their views regarding adopting selfBACK in this context. Furthermore, patients were most likely interviewed when they already received first consultation and initiated treatment in specialist care (ie, 3-4 months after inclusion), and this might have affected their views on self-management and the use of digital interventions. Finally, while we interviewed patients with different app usage levels, we did not interview patients who had never accessed it. This could have provided better insight into factors related to the onboarding procedure and uptake of digital interventions in this setting.

### Conclusions

Both patients and health care practitioners supported the adoption of app-based self-management support for low back and neck pain in specialist care. The selfback app was reported by some patients and health care practitioners to provide reassurance and empowering patients to take actions for their health problem on their own. Acceptance and engagement with the app-based intervention can be influenced by various factors, such as content structure and relevance, tailoring, trust, and usability properties. Digital self-help combined with human support might be necessary to enhance adoption of self-management, particularly in specialist health care settings.

## Abbreviations

AI: artificial intelligence
COREQ: consolidated criteria for reporting qualitative research
RCT: randomized clinical trial
